# My voyage in the enchanted world of sleep

**DOI:** 10.1093/sleepadvances/zpae027

**Published:** 2024-05-17

**Authors:** Peretz Lavie

**Affiliations:** Ruth and Bruce Rappaport Faculty of Medicine, Technion – Israel Institute of Technology, Haifa, Israel

## Abstract

In this paper, I describe my 45-year career in sleep research. I started my undergraduate studies at Tel Aviv University, where I was first introduced to the enchanted world of sleep, continued to my graduate studies with Wilse B. Webb at the University of Florida, and then to post-doctoral training with Dan Kripke at the University of California at San Diego. Then, I describe the evolution of my academic career at the Technion-Israel Institute of Technology, where I started in 1975 as an Assistant Professor and retired in 2019 as the President of the Institute. I describe the areas of research that I pursued and how the research developed, emphasizing unexpected results that guided me and my lab team in new directions. This includes my early studies on ultradian rhythms, inspired by Nathaniel Kleitman’s Basic Rest Activity Cyle hypothesis, utilizing the ultrashort sleep–wake paradigm to chart the 24-hour sleep propensity function, and how these studies led us to explore the role of melatonin in sleep regulation. I also explain why we directed our attention to sleep apnea, and how clinical observations led to the provocative hypothesis that sleep apnea—typically seen as a disorder—may also play a protective role. Under the leadership of my research partner and wife, Lena, we confirmed this hypothesis. Also in this article, I describe my enthusiasm for the history of our field and, as derived from my experience as a Dean of Medicine and President of a university, I share my philosophy about the role of members of academia in society. I emphasize that none of my achievements could have been accomplished without the hard work and motivation of my students and research partners, who shared my enthusiasm and passion for the enchanted world of sleep.

This paper is part of the Living Legends in Sleep Research series, which is sponsored by Idorsia Pharmaceuticals and Jazz Pharmaceuticals.

## First Steps

How did I get into sleep research? In 1969, when I was an undergraduate student in the Department of Psychology at Tel Aviv University (TAU), Prof. Zvi Giora, who had an interest in dreams, convinced the Department to open a one-bedroom sleep laboratory. Its purpose was to identify REM sleep in order to collect dreams. To my good fortune and purely by chance, I was given the opportunity of making a little extra money as a sleep technician by overseeing the night recordings. With the benefit of hindsight, I can now say that it was that opportunity, which sealed my fate as a sleep researcher. Years later, when I asked Prof. Giora why he approached me, his answer was, “I picked the student who got the highest grades in physiological psychology.” Indeed, this was my most favorite course, taught by an intellectual giant, Prof. Yeshayahu Leibovitz from the Hebrew University of Jerusalem. This is how I started my voyage in sleep research, spending many sleepless nights overseeing the ink-writing pens of an ancient Galileo electroencephalograph, carefully searching for the signs of REM sleep, and then waking up the participant to record his dreams. Often, in the small hours of the night, I would willingly change places with the participant sleeping soundly in the examination room.

I must admit that when starting my night job, I knew nothing about sleep. In fact, when I decided to study psychology at TAU, my intention was to specialize in social psychology, because I had a longtime interest in education. But the magic of identifying the precise moment of dreaming fascinated me. I felt rather unique; in 1968, the field of sleep research was in its infancy—essentially *terra incognita*. No one in the Department knew anything about sleep, and the new sleep laboratory attracted the attention of students and faculty alike. To learn more about the field, I spent time in the library reading all I could find about sleep research. In that year, only 500 scientific papers related to human sleep had been published, and most papers were simply titled “Sleep,” “Loss of sleep,” or “Circadian rhythms” give the newness of it all. I was fascinated by the writings of Nathaniel Kleitman and William C. Dement, two of the giants of the field whom I was lucky enough to get to know later in my career. While Kleitman’s 1953 *Science* paper, coauthored with his student Eugene Aserinsky, was a must-read for anyone interested in sleep, I was particularly fascinated by his hypothesis of the existence of the Basic Rest Activity Cyle (BRAC), a basic periodicity spanning 50–60 minutes in infants and increasing to 80–90 minutes in adults, which appears as a series of REM episodes in advanced sleep and as recurrent fluctuations in alertness in advanced wakefulness.

In the landmark CIBA Foundation symposium (1961), on The Nature of Sleep **[1]**, Kleitman concluded his presentation, entitled *The Nature of Dreaming,* by speculating that: “The regular succession, at 80–90 minute intervals, of periods of emergent stage 1 EEG, with REM’s and dreaming, is perhaps related to a primitive rest-activity cycle reported in a study of sleep characteristics of infants ……..This cycle is obscured during wakefulness by the great surge of cortical activity, but suggestions of its presence may be discerned in daytime oscillations in alertness, the often irresistible drowsiness after a big meal, and the relief that some persons get from brief cat-naps.” (pp. 360-361). He repeated his suggestion in the second edition of *Sleep and Wakefulness* published in 1963 **[2]**, (p. 365). In another paper, he encouraged researchers to: *“*Study wakefulness. Study the rest-activity cycle. Don’t just study sleep*”***[3]**. Dement, who joined Kleitman’s laboratory shortly after the discovery of REM sleep, focused his attention in the 60s on demonstrating that REM sleep is indeed dream sleep. In a series of studies, he meticulously investigated dream reports after waking up from REM sleep **[4]**. Dement’s studies were invaluable to a newcomer like me, in my many sleepless nights in the laboratory collecting dreams.

In my work meticulously waking up sleepers from REM sleep, I decided to test Kleitman’s BRAC hypothesis by comparing arousal after waking up from REM sleep and non-REM sleep. I reasoned that if the REM-non-REM cycle is only the sleep portion of Kleitman’s BRAC, then there will be carry-over effects from the two stages of sleep to wakefulness. At that time, I found only two studies that compared the effects of awakening from REM and non-REM sleep. A projective personality test (Thematic Apperception Test) elicited more vivid and talkative responses when waking up from REM compared to non-REM sleep [5]; and visual evoked potentials in post-REM awakenings were highly similar to those in the pre-sleep–wakefulness period, but dissimilar, both in amplitude and in latency, in post-non-REM awakenings [6]. To measure the level of post-awakening arousal I used the duration of an illusion, which was shown to reflect the level of cortical arousal (the spiral after effect, SAE). I predicted longer durations after waking up from REM sleep. To my delight, the results confirmed my hypothesis. The illusion was significantly longer after waking up from REM than non-REM sleep **[7]**. I still remember the excitement I felt when my first paper was accepted for publication. No doubt, the experience of performing an independent study and being able to publish my first paper, as an undergraduate student, sealed my decision to continue my education in sleep research.

After graduation from TAU, I won a scholarship that allowed me to continue my studies in the United States. I spent 1971 at Florida State University (FSU) in Tallahassee, where I completed an MA degree. Since no one in the FSU Department of Psychology had anything to do with sleep research, I had to organize my own laboratory. Prof. Lloyd Elfner, who studied the auditory system, kindly allowed me to use some of his soundproof rooms, to which I brought an old electroencephalograph that I borrowed from a different laboratory. I found a supervisor 100 miles away. I called Wilse Bernie Webb at the University of Florida (UF) in Gainesville, told him about my earlier study and what I plan to do, and asked him to chair my master’s degree committee. To my delight, Webb, one of the founders of sleep research, agreed. I managed to complete my MA degree within a year. I replicated my earlier SAE findings demonstrating that the differential post-awakenings effects persist for at least 15 minutes **[8]**. Even more importantly, on that year, on Thanksgiving, I married Lena, who had graduated from Biology at Ben-Gurion University in Israel and joined me in Tallahassee.

I was delighted that after completing my degree, Webb offered me the chance to join the UF PhD program in physiological psychology, and more importantly, a position as his graduate research assistant. My years in Webb’s laboratory were very productive and educational, although, as in FSU, I had to build my own sleep laboratory, and conduct research with “zero budget.” Joining Webb’s group, I planned to continue investigating the BRAC hypothesis, but unfortunately, I could not use the then famous Webb’s sleep laboratory. Since the two-bedroom sleep laboratory was dedicated to long-term studies of sleep in a time-free environment, I had to run my studies elsewhere. Another reason was that Bob Agnew, Webb’s life-long research partner, was very possessive about the use of the laboratory. He did not allow any of Webb’s students to interfere with the ongoing studies.

Thus, I found myself, again, forced to set up my own laboratory. I managed to a find an empty space in the Department of Psychology, borrowed an electroencephalograph from another laboratory, and recruited volunteers for my studies from local high schools, where I gave free talks about sleep. Since some of the studies lasted for more than 24 hours and I needed assistants, I convinced some of my colleagues to join me in return for authorship in my publications. In a span of 2 years, I managed to complete my PhD studies. First, I expanded my studies comparing REM and non-REM post-awakening arousal using an optical illusion known as the beta movement, or the phi phenomenon, in which two proximal lights alternately flashing on and off in a specific frequency range are viewed as apparent motion [9]. The range of frequencies for which participants see the apparent motion is a sensitive index of brain arousal. As hypothesized, the frequency range in which participants reported apparent motion was wider after REM than after non-REM sleep.

I concluded these studies by demonstrating the existence of 80–100 minutes REM-non-REM-like cycles during wakefulness in the duration of both optical illusions (the SAE and the beta movement), which I interpreted as supporting Kleitman’s BRAC hypothesis [10, 11]. As promised, some of my assistants received authorship. Meanwhile, Webb, who was the chairman of my PhD committee, and made helpful suggestions to my papers, felt that, given the circumstances, he could not add his name. To me, this was a good lesson about principles of authorship. However, we jointly published a single paper on time perception in a time-free environment. We showed that the tendency to underestimate the passage of time during time-free isolation is primarily determined by behavioral cues [12].

After graduation in 1974, I moved westward, from Florida to California. I was offered a postdoc position in Daniel Kripke’s laboratory in the Department of Psychiatry at the University of California-San Diego. Dan had trained with Eliot Weitzman in New York, one of the pioneers of sleep research, and moved to San Diego in 1971, where he opened his own sleep lab in the VA Hospital. I decided to join Dan because of his interest in ultradian rhythms. In 1972, Dan published a paper on REM-non-REM-like ultradian rhythms in EEG activity and several behavioral and physiologic systems **[13]**, which caught my attention. This convinced me that I would find common language with Dan, and I was not mistaken. The drive from Florida to California was a once-in-a-lifetime experience: we drove in a 1967 Dodge Dart, with all our belongings plus a tent that served us during the nights and visited some of the national parks along the way.

My postdoc position in San Diego was supported by a grant from a drug company. The research focused on the complexities of insomnia, and I was exposed to the intricate world of rivalry and competing pharma companies. The drug project left me enough time to continue my investigation of Kleitman’s BRAC hypothesis. In one of these studies, we confirmed a report published at the turn of the 20th century on the existence of 90-minute cycle in gastric contractions in sleeping humans [14]. However, unlike the BRAC hypothesis, the gastric cycle was independent of the REM-non-REM cycle **[15]**. A paper in *Science* on ultradian rhythms in urine flow in sleeping catheterized patients, synchronized with the REM-non-REM cycle **[16]**, prompted us to search for such cycles during waking. To avoid catheterization, participants were required to drink a constant amount of fluid and then to urinate, as much as possible, every 10 min for 10 hours. Remarkably, 17 out of the 20 participants completed the study. Most of them revealed distinct 100-minute periodicity in the amounts of urine that was out of phase with similar rhythms in urinary osmolality, and electrolyte concentrations **[17]**. These findings were so robust that we decided to submit the paper to *Nature*. Surprisingly, it was accepted, as is, in record time. A few years later I met Jürgen Aschoff, a giant in circadian rhythms research from Germany, who told me that he reviewed the urine flow paper. He said he was astonished by the results and was so impressed by the idea and resilience of the participants that he recommended immediate publication. However, despite the paper’s publication in such a distinguished journal, ultradian rhythms in urine flow did not impress the biological rhythms community. Since its publication, it has been cited only 54 times.

Our stay in beautiful San Diego was marked by two outstanding events. First, Karen, our first daughter, was born a day after our Thanksgiving anniversary, in Scripps Memorial Hospital. Soon after, Lena started working in Roger Guillemin’s laboratory at the Salk Institute, which gave us more financial freedom. Three years later, in 1977, Guillemin won the Nobel Prize in Physiology and Medicine for his work on neurohormones.

The second highlight was an event that left a marked impression on the entire staff of the sleep laboratory. In retrospect, it was a prologue to the emerging field of sleep medicine. One day, the chairman of the Department of Internal Medicine called Dan to consult with him about a patient whose loud, intolerable snoring disturbed the sleep of the entire department. He also mentioned that the patient’s sleep was very restless, and that during the day he spent most of the time asleep. Since very few in the Veteran’s Administration (VA) hospital were aware of the existence of a sleep laboratory, Dan cleverly seized the opportunity to publicize the lab. He suggested taking the patient over to the sleep lab for an afternoon nap. I vividly remember the event: the chairman and his residents, standing in a small bedroom around the patient’s bed, watching me attach the electrodes for sleep recordings. The patient, an obese man in his 50s, fell asleep during the preparation. Once asleep, he immediately started struggling for air, experiencing 40- to 50-second-long apneas. Each was terminated by violent body movements and loud gasping and snoring. The struggle for air alarmed the physicians who watched the sight with astonishment. One of them, standing next to me, insisted that I should wake up the patient “because he may die in his sleep.” I had to assure him that he would not die, “because this is the way he has been sleeping every night for many years.” It was not surprising that none of the physicians who witnessed the event had any knowledge about sleep apnea; in 1974, there were only a handful of papers on the subject, mostly from Stanford, linking apneas during sleep to insomnia, as well as from Elio Lugaresi’s group in Bologna, Italy. At the time of this incident, it did not cross my mind that I would see tens of thousands of similar patients in coming years.

In the middle of 1974, my life took another turn. I received a telephone call from the Technion-Israel Institute of Technology in Haifa, offering me a lecturer (Assistant Professor) position. My first reaction was that someone made a mistake, since the Technion is a technical university, and I am a psychologist. But then I was assured that the offer was indeed intended for me, and the position would be in the newly established Faculty of Medicine. It took me 2 seconds to agree, and a few months later, the three of us returned to Israel. On our way to Israel, we made a stopover in Edinburgh, Scotland, to attend one of the first international sleep meetings. This was also my first-ever presentation at a scientific meeting. I gave a 10-minute talk on the secretion of parathyroid hormone (PTH) during sleep. Disappointingly, there were only 10 people in the audience **[18]**.

## My Own Sleep Lab

When I joined the Technion in 1975, the Faculty of Medicine was housed in an old building that used to be a monastery, just across from RAMBAM Hospital, near the port of Haifa. Since there was no appropriate place for a laboratory in that building, I was provided a space on Technion’s main campus, on the slope of Carmel Mountain, a 20-minute drive from the faculty. In retrospect, being a part of the main campus with its thousands of students was a great advantage. The faculty provided me with a modest budget that allowed me to open a four-bedroom sleep laboratory equipped with a single electroencephalograph. My first years at the Technion were dedicated to establishing the sleep laboratory as a vibrant and active research lab ready for collaboration with almost anybody who had an interest in sleep. I also spent time attracting graduate students and medical students and sharing my enthusiasm for sleep research to expand the field. Thus, in my early studies, I collaborated with different clinical departments, studying and publishing on the sleep of patients with Parkinson’s disease **[19]**, depression **[20]**, Kleine-Levin syndrome **[21]**, and familial dysautonomia **[22]** patients, among others.

In parallel to these collaborative studies, we started a program to investigate ultradian rhythms in different physiological systems. Encouraged by our findings on ultradian rhythms in urine flow, I decided to better understand this phenomenon in a canine model. Dr. Carlos Gordon, a PhD student in my lab, found ultradian rhythms in urine flow that were synchronized with the dog’s sleep–wake cycle but not with the dog’s REM-non-REM cycle **[23]**. Later, we reported that the rhythms in urine flow were independent of episodic secretion of anti-diuretic hormone **[24]**. We also conducted a series of studies on ultradian rhythms in a variety of arousal indices such as pupillary response to light **[25]**, motor task performance **[26]**, and density of EEG alpha rhythms **[27]**. I concluded this chapter in my research career with the understanding that ultradian rhythms in a variety of physiologic and behavioral systems reflect a multi-oscillatory phenomenon, a sort of “biological hour” and not a single BRAC, as originally suggested by Kleitman **[28]**.

## The 24-Hour Sleep Propensity Function

Even though we did not confirm the existence of Kleitman’s BRAC, we found evidence that arousal during the waking portion of the 24-hour fluctuates with periodicities like the REM-non-REM cycle. Since we received a grant from Israel’s Ministry of Labor to investigate the influence of daytime sleepiness on safety at work, I was looking for a reliable way to measure fluctuations in sleepiness throughout the 24-hour cycle. As I will describe later, the same grant also played a crucial role in my growing interest in sleep apnea syndrome. The idea of how to reliably chart the 24-hour fluctuations in sleepiness came to me after reading papers by Carskadon and Dement on the 90-minute day.

In two papers published in 1975 and 1977 [29, 30], they reported that participants placed on a 90-minute sleep–wakefulness schedule for 5 or 6 days, during which they were permitted to sleep for 30 minutes, separated by 60 minutes of enforced wakefulness, showed frequent sleep-onset REM periods that alternated with slow wave sleep (SWS) periods. Increased subjective sleepiness was correlated with SWS and decreased sleepiness with REM sleep. Impressed by the ability of participants to adapt to the 90-minute “day” for 6 days, I decided to shorten the “day” to 20 minutes, allowing 5 minutes of sleep alternating with 15 minutes of enforced wakefulness. This resulted in a napping frequency that is sensitive to ultradian variations.

The first experiment using the 5/15 ultrashort sleep–wake protocol was conducted for only 12 hours, after a night of normal sleep, a night of selective REM deprivation, and a night of total sleep deprivation. Analyzing the occurrence of sleep stage 1 in each one of the 36 5-minute sleep opportunities, revealed approximately 100-minute periodicity, thus, the appearance of stage 1 occurred in sleep attempts spaced approximately 100 minutes apart. In contrast, the occurrence of stage 2 appeared mostly in the afternoon period. REM and SWS rarely occurred. Surprisingly, despite the progressive accumulation of sleep deprivation, the amount of sleep decreased during the evening hours. Both selective REM deprivation and total sleep deprivation modified this pattern and increased the amounts of sleep stage 2 in each of the sleep attempts **[31]**. In the second study utilizing this paradigm, we studied the phase relationship between the REM-non-REM cycle and the cycles in sleep propensity during waking hours. In this experiment participants began a 5/15 schedule at 16:00 until 24:00, then they retired for uninterrupted nocturnal sleep. They were awakened after 6-7 hours, either from REM sleep (in one experimental period) or 25 minutes after the end of REM sleep (in a second experimental period), and a second 5/15 schedule was initiated for 8 hours. In the REM awakening condition, synchronizing the individual time series constructed from the occurrence of stage 1 to the last nocturnal REM period, revealed a significant ultradian 100-minute sleep propensity cycle, which was most prominent during the first 4 hours after awakening. Also, there was a tendency toward higher levels of sleepiness during the first hour after awakening from non-REM sleep, than after awakenings from REM **[32]**.

While the first two publications on ultradian cycles in the occurrence of stage 1 did not arise much interest in the field, my third ultrashort sleep–wake study attracted a lot of attention and was cited so far 682 times **[33]**. Encouraged by the ability of participants to adapt to the 5/15 schedule for 12 hours, we modified it to the 7/13 schedule and extended it to 24 hours. This comprised of three experiments, with and without prior sleep deprivation. In addition, we investigated the effects of contrasting experimental demands: instructing participants to attempt to sleep for 7 minutes, or conversely, instructing them to resist sleep for 7 minutes. As could be expected, there were significant differences in the total amount of sleep in the “attempting” and “resisting” sleep conditions, but the temporal structure of sleep propensity was remarkably similar. In each experiment, there was a major nocturnal sleep period consisting of 20–25 consecutive 7-minute trials with at least 5 minutes of sleep in each, and a secondary mid-afternoon sleepiness peak, at around 16:00.

The most striking results were that the onset of the nocturnal sleep period was abrupt, almost an “all-or-none” phenomenon, and that this “sleep gate” was preceded by a pronounced decrease in sleep propensity. That is, just before the sleep gate, participants were unable to fall asleep when instructed to do so and could easily resist sleep when requested to remain awake. This happened despite progressive sleep deprivation. We termed this early evening period of decreased sleepiness “the forbidden zone for sleep.” The timing of the sleep gate was a stable individual characteristic, which was later confirmed in a study comparing the 24-hour sleep propensity pattern of “morning” and “evening” persons **[34]**. Later, by extending the 7/13 paradigm to 48 hours, we showed that not only does the temporal structure of sleep propensity have a remarkable individual consistency, but the overall level of sleepiness does as well. We suggested that based on these results, people can be categorized into “sleepy” and “alert” somno-types **[35]**. The stability of the sleep gate supported the accumulated evidence that the morning and evening typology is genetically determined. The suggested somno-typology for “sleepy” and “alert” individuals still awaits further research.

Two studies performed by my PhD students, Orna Tzishinsky and Tamar Shochat, demonstrated that the timing of the sleep gate was phase-locked to the onset of nocturnal melatonin secretion. The increase in melatonin preceded the sleep gate by 100 to 120 minutes **[36, 37]**. Studies also showed that evening exposure to 2 hours of bright light significantly delayed the next-day sleep gate while early evening administration of exogenous melatonin advanced the sleep gate **[38, 39]**.

The ultrashort sleep–wake cycle has been widely used in my laboratory. Many of my graduate students have spent prolonged hours supervising these studies, particularly ensuring that sleep-deprived participants will not fall asleep during the 13-minute wake portion of the 20-minute “day.” Based on the temporal structure of REM occurrences in the 7/13 paradigm, we showed that the first appearance of REM in the 7/13 paradigm is dependent on a critical accumulation of non-REM sleep, but once activated, it continues to periodically appear, regardless of accumulated non-REM sleep **[40]**. Similarly, tested with the same paradigm, untreated narcoleptic patients showed a 70- to 80-minute cycle in the occurrence of REM periods, independently of accumulated non-REM sleep **[41]**. Older adults tested with this paradigm showed a tendency toward lower sleepiness peaks and advanced sleep gates **[42]**. Combining the 7/13 paradigm with two nap conditions, one at the time of the midafternoon sleepiness peak, and second, at the time of the forbidden zone for sleep, revealed that the midafternoon nap was more efficient, and produced less sleep inertia than the late nap **[43]**.

The ultrashort sleep–wake paradigm was very demanding, both for the participants and laboratory staff, and has provided important information on the 24-hour temporal structure of sleep propensity. At a time when the literature was dominated by the two-process sleep–wake model, which posited the existence of a sleep–wake-dependent homeostatic process (Process S) that regulates sleep by interacting with a process controlled by the circadian pacemaker (Process C) **[44]**, there was no place for a “forbidden zone” for sleep. Its existence in a sleep-deprivation condition contradicts the homeostatic principle. However, it was gratifying that later studies, using different methodologies, confirmed the existence of a forbidden zone for sleep **[45, 46]**, although in these studies it was termed the “wake maintenance zone.”

Several chapters in books and a review in the *Annual Reviews of Psychology***[47]** provide comprehensive summaries of our studies **[48]**. I must admit, however, that in hindsight, these studies had a major shortcoming, regretfully, which is that we investigated only males. At that time, males comprised most of the student body at the Technion. Furthermore, due to the highly sophisticated study protocols and repeated measures design, studies were limited in sample size; thus, we were concerned that including female participants would introduce variability and decrease statistical power.

## Melatonin—A Soporific Hormone

The finding that the sleep gate is phase-locked to the nocturnal increase in melatonin secretion directed our attention to the role of melatonin in sleep–wake regulation. Between 1991 and 2005, we published 42 papers on various aspects of melatonin secretion. Orna Tzishinsky, who showed that bright light and melatonin administration can shift the timing of the sleep gate, also showed that in blind children delayed secretory peaks in melatonin were significantly associated with disturbed sleep and with increased fatigue and napping during the day **[49]**. Decreased circulating melatonin secretion in aging, and its association with insomnia, was the subject of Iris Haimov’s PhD dissertation. In collaboration with Prof. Nava Zisapel from TAU, Iris showed that deficiency in melatonin secretion and disruption of its rhythms were associated with increased prevalence of sleep disorders in older adults **[50]**. In a subsequent study, Iris showed that using a low dosage of sustained and fast-released melatonin, administered two hours before bedtime, significantly improved sleep initiation and maintenance in older adults with insomnia who had low levels of melatonin **[51]**. This study, demonstrating the efficacy of melatonin replacement, was the basis for the development of a therapeutic formulation of sustained-release melatonin (Circadin™), by Prof. Zisapel. Melatonin replacement therapy also restored sleep continuity in a child with a pineal tumor that dramatically suppressed melatonin secretion, associated with severe insomnia **[52]**. Administration of 3 mg melatonin to melatonin-deficient children with psycho-motor retardation who had irregular sleep–wake patterns also significantly improved their sleep **[53]**. Based on our accumulated findings we proposed that melatonin is a soporific hormone that contributes to the induction of sleep by an inhibition of a wakefulness-producing mechanism **[54]**.

The role of melatonin in regulating animals’ reproduction and its increasing use in humans, prompted us to conduct a series of studies to examine if it also affects hormones involved in human reproduction. All these studies were conducted in the sleep laboratory, concomitantly with sleep recordings. In collaboration with the endocrinologist Rafael Luboshitzky, we found hypersecretion of nocturnal melatonin in males with delayed puberty, because of Gonadotropin-releasing hormone (GnRH) deficiency **[55]**. In contrast, there was decreased nocturnal melatonin secretion in Klinefelter syndrome patients with hypergonadotropic hypogonadism **[56]**. Treatment with testosterone normalized melatonin secretion in both patient groups **[57]**, suggesting that both sex steroids and gonadotropins modulate pineal melatonin secretion. Despite these findings suggesting a role of melatonin in human reproduction, long-term daily melatonin administration to adult men had no effect on gonaodotrophin-gonadal steroid hormone secretory patterns **[58]**, although we found hints that it may alter semen quality **[59]**. A summary of these studies was published **[60]**.

## Sleep Apnea—The Impetus for Sleep Medicine!

Although patients struggling for air during sleep were described as early as the 18th century, scientific research on sleep apnea with an eye toward potential treatment was slow in coming. We began examining people complaining of sleep-related problems at the Technion’s sleep lab toward the end of the 1970s. In opening the service to the public, we were sure that we would see mostly patients with insomnia and narcolepsy, but surprisingly, most of the people who approached us complained of excessive daytime sleepiness (EDS).

At that time, EDS was considered relatively rare in the general population. This prompted us to investigate its prevalence in Israel. To do that, we interviewed a random sample of 1502 industrial day-laborers in 250 factories about their sleeping habits, sleep disturbances, work conditions, medication use, and general health. Then, we performed laboratory sleep recordings in three groups: 41 workers with no sleep complaints; 17 complaining of insomnia; and 20 complaining of EDS. The results were surprising: 20.5% of the non-complainers, 40% of the EDS group, and 11.7% of the insomniacs, had an apnea index (AI) > 5; 14.1% of all workers, and 35% of the EDS group, had AI > 10. I should add that only apneas ≥ 10 seconds were counted, since at that time we were unaware of the importance of hypopneas. Using AI > 10 as a cutoff point, plus typical complaints, we estimated that at least 1% of men older than 21, and 4% of men older than 40, experiences sleep apnea **[61–63]**. Also, workers with AI > 10 reported significantly more instances of hypertension. I concluded that paper with the following prediction: *“*The present data suggest that for a rather large segment of the adult male population, diagnostic poly-hypnographic recordings are an essential diagnostic procedure.”

I presented our findings on the prevalence of sleep apnea in Israel for the first time at the 20th annual meeting of the APSS in Mexico City, in 1980. At the end of my presentation, a prominent sleep researcher cynically commented, “It would appear from your presentation that breathing disorders during sleep are particularly prevalent in the Middle East.” He did not believe that so many men experienced sleep apnea; in 1980, the emerging field of sleep medicine was not ready to accept the fact that sleep apnea affects so many people. Terry Young’s landmark publication in the *NEJM* was the turning point [64]. Young et al (1993) estimated that 4% of middle-aged men experienced sleep apnea, an estimate identical to ours made over 10 years earlier. While it was reassuring that sleep apnea was not unique to the Middle East, it also proves that to convince the medical community you should publish in the *NEJM*.

The opening of the Technion Sleep Clinic, which was the first of its kind in Israel, in addition to the sleep research laboratory, proved to be an invaluable source of clinical materials and research ideas. When we started seeing patients, one could obtain the impression that only the residents of Haifa and its environs experienced sleep apnea, because we did not see patients from other parts of the country. However, when in 1985 and 1990, we opened clinics in Tel Aviv and Jerusalem, it became clear that these cities have their share of sleep apnea. Based on data that began accumulating in these clinics, we confirmed our initial observation on the association between sleep apnea and hypertension **[65]**. Using ambulatory 24-hour blood pressure monitoring, we showed that the level of 24-hour blood pressure was significantly related to the syndrome severity **[66]**. Later, in collaboration with Victor Hoffstein from Toronto, we confirmed, in a large group of sleep clinic patients, that the risk of hypertension is dependent on apnea severity **[67]**. Prompted by the observation that apneic industrial workers had significantly more ENT problems, we conducted several studies to investigate the importance of nasal breathing in sleep. We demonstrated that mechanical nasal obstruction for only one night caused a significant increase in the number of apneas **[68]**, particularly in offsprings of sleep apnea patients **[69]**. These results were later confirmed and expanded by Giora Pillar’s PhD studies **[70, 71]**. Collaborating with the Department of Surgery at Soroka Medical Center in Beer Sheva, that pioneered gastric bypass surgery for massive obesity, we documented dramatic post-operational decrease in the severity of sleep apnea, concomitant with post-surgery weight reduction **[72–74]**.

One of the clinical observations that caught my eye was a group of 80 to 90-year-old patients all having severe sleep apnea, who, except for EDS, did not have any significant co-morbidities. These prompted me to investigate the mortality of sleep apnea patients in a systematic way. The large number of patients who spent nights in our clinics, and the existence of a national registrar on mortality across different age groups, allowed us to do it. My term as Dean of Medicine between 2003 and 2009 most probably also helped me acquire the data needed for such studies.

Our mortality studies yielded surprising results: the risk of mortality in patients with sleep apnea was high for young patients but declined with age **[75, 76]**. Moreover, the risk of mortality not only declined with age, but we also found unexpected significant survival advantages in elderly people with sleep apnea, suggesting that the syndrome played a protective role **[77]**! These findings explained my clinical observations of the “healthy” 90-year-old sleep apnea patients. Yet it was very difficult to publish these results in leading journals. The idea that sleep apnea may be protective was provocative; it contradicted the unanimous agreement in the literature that it is a major risk factor for cardiovascular morbidities, diabetes, stroke, and consequently mortality. This belief fueled many large-scale studies financed by generous grants.

**Figure 1.**
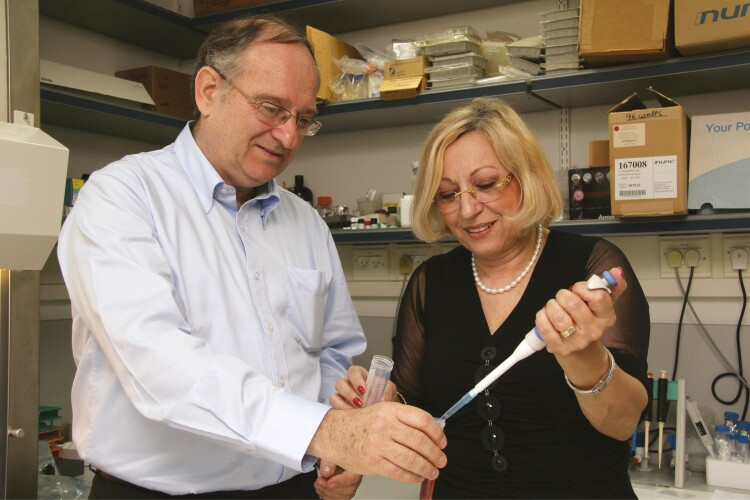
Sleep apnea research became a family affair—with Lena in 2009 (Credit: Yoav Bachar, Technion—Israel Institute of Technology). For one-time usage ONLY. Not to be transmitted.

Understanding the impact of sleep apnea on the cardiovascular system and the possible activation of protective mechanisms required knowledge and expertise that I did not have. Fortunately, I had it at home. My wife, Lena, who after our return from San Diego, completed her PhD at the Technion’s Department of Biology in the field of cell biology, was an expert in inflammation and oxidative stress. Lena was a researcher in the Faculty of Medicine, thus, in the late 90s, sleep apnea research became a “family affair.” (Figure 1)

Our group was joined by Larissa Dyugovskaya, an immunologist who had come to Israel from the former Soviet Union. This collaboration proved to be very successful, producing several breakthrough discoveries and 42 joint scientific publications. Since I was busy in the Dean’s Office and later as President of the Technion, Lena ran our research group. She demonstrated that intermittent hypoxia, the hallmark of sleep apnea, produces free radicals and oxidative stress **[78]**, which in turn activates adhesion molecules **[79–82]** that initiate endothelial dysfunction. This cascade of events results in atherosclerosis in the arteries, hypertension, and heart disease. I vividly remember her first presentation on sleep apnea as an oxidative stress disease. It was at the 1999 World Federation Sleep meeting, in Dresden, Germany. At the conclusion of her talk, a well-known researcher who worked on hypertension in sleep apnea, stood up and asked her in a very patronizing way, “Dear lady, do you know what you are talking about?” I am convinced that he changed his mind after reading her seminal paper, “Sleep apnea as an oxidative stress disease*”***[83]**, which is one of the 10 most-cited papers in *Sleep Medicine Reviews*.

Our unexpected findings on age-related decline in mortality and the survival advantage in sleep apnea elderly patients were puzzling, to say the least. We hypothesized that these results may be explained by a phenomenon called ischemic preconditioning **[84]**. This is a state in which repeated sublethal ischemia confers profound protection from infarction, arrhythmia, and further ischemic insults **[85]**. Although ischemic preconditioning is elicited largely by exposure to a few sublethal ischemic events, we hypothesized that the nocturnal intermittent hypoxia similarly confers cardio-protection; furthermore, we found support to our hypothesis by the fact that free radicals produced in sleep apnea were implicated in activating ischemic preconditioning.

To test the activation of adaptive pathways in sleep apnea, Slava Berger, our PhD student under Lena’s supervision, compared the level and functions of endothelial progenitor cells (EPC) in patients with acute myocardial infarction (AMI) and sleep apnea, and patients with AMI but without sleep apnea **[86]**. The results were clear-cut: EPC numbers and proliferative and angiogenic properties are heightened in patients with AMI with sleep apnea. Furthermore, EPCs from healthy individuals exposed to intermittent hypoxia in vitro behaved the same, implicating the role of intermittent hypoxia in augmenting EPC numbers and functions.

Since the publication of this paper, a large number of studies confirmed the protecting properties of sleep apnea in a variety of clinical conditions. These include obesity-hypoventilation syndrome, in-hospital mortality of post-surgery patients, in-hospital patients hospitalized for different reasons, patients with pneumonia, patients in the intensive care unit and patients hospitalized for myocardial infarction, patients hospitalized for pulmonary embolism, and critically ill patients. In all these conditions, patients with co-existing sleep apnea showed survival advantage **[87–91]**. I summarized some of our extensive research on sleep apnea and our vast experience with the diagnosis and treatment of sleep apnea, in the book*: Restless Nights: Understanding Snoring and Sleep Apnea*, published by Yale University Press in 2003 **[92]**.

## Trauma and Sleep—What a Nightmare!

In Israel, a country with a history of so many wars, it is not surprising that post-traumatic stress disorder patients (PTSD) were often seen in our sleep clinic. Some were veterans of the Six-Day War in 1967 and the Yom Kippur War in 1973. Others had been traumatized as victims of terrorist attacks, and some were Holocaust survivors. Although all were referred to the clinic because of complaints of sleep disorders, the sleep laboratory findings were not uniform. In some individuals, we found difficulties falling asleep, mid-sleep awakenings, and frequent nightmares—all things we would expect to find—but in many others, despite having the same complaints, sleep was intact **[93]**. Similarly, we found a discrepancy between subjective complaints and PSG findings in traumatized victims of traffic accidents **[94]**. To better characterize sleep in traumatized patients, we compared sleep of PTSD patients exposed to different types of trauma with age-matched controls. Our initial results were surprising. While some of the patients indeed had PSG-documented insomnia, and REM and non-REM-related nightmares, in many others, in spite of similar complaints, we found very low dream recall **[95]**. In seven patients awakened 21 times from REM sleep, there were only four dream reports with some content. In 17 awakenings, they denied dreaming.

Dr. Hana Kaminer, a PhD student trained in clinical psychology, took it upon herself to examine dreaming in traumatized patients. Hana studied Holocaust survivors who were well-adjusted to life after the Holocaust, Holocaust survivors who were less adjusted to life, and age-matched controls. The division of survivors into well- and less-adjusted was based on clinical interviews and questionnaires. The results were surprising. Well adjusted survivors had the lowest dream recall after awakenings from REM sleep ever reported in the dream literature (33.7%). This was significantly less than the less adjusted (50.5%) and controls (80%).

Like in other war-affected patients, in most cases, well-adjusted survivors whose sleep was intact denied dreaming. The few dreams they remembered were brief “telegraphic” dreams, containing few words (a sentence or two), without emotions or narrative, while half of the dreams of the less adjusted survivors were anxiety dreams **[96, 97]**. Later, in his PhD dissertation, Yaron Dagan, another student in our lab, showed that waking thresholds in war-related PTSD patients were higher than controls, even though their sleep quality was no different than controls **[98, 99]**. We suggested that suppression of dreaming, perhaps by deepening sleep, is one of the mechanisms enabling long-term adaptation to traumatic events. This interpretation—which contradicted the prevailing view that adapting to traumatic events is accomplished by “mastering the trauma”—made it difficult to publish the results in leading Journals. Psychiatrists and clinical psychologists found it difficult to accept the idea that repression can serve an adaptive function. I became keenly aware of this attitude from the responses to my keynote presentation at the Annual Meeting of the Israeli Psychiatric Association. However, there were some gratifying forms of compensation. I was invited by the editor of the *NEJM* to publish a comprehensive review of our findings, which was entitled “Sleep Disturbances in the wake of Traumatic Events” **[100]**, and the BBC in London produced a documentary on Hana Kaminer’s Holocaust survivors’ study, entitled “To sleep perchance to dream.” In view of the attacks of October 7, 2023, which were highly traumatic events in Israel, which have affected a large number of children and adults, our observations on the protective role of repression may be of importance regarding their long-term adaptation.

## Fascinated by History

Often, I am asked: “What would you have done if you had not become a sleep researcher?” To which I answer without hesitation: “A historian.” Since my high school days, I have been fascinated by history. When I joined the field of sleep research, one of my pastimes was to search for, and read, ancient books and publications about sleep, sleep disorders, and biological rhythms. I had standing orders with some secondhand and old book dealers to alert me whenever they came across such a book. I have quite a collection of them. I was fascinated by the lives and careers of people like Richard Katon **[101]**, Constantin von Economo **[102]**, Thomas Laycock, and Edward Smith **[103]**, who, relying only on their sharp eyes and clinical acumen, made original insights concerning brain activity, sleep, and origin and meaning of biological rhythms. Interestingly, the first edition of Kleitman’s *Sleep and Wakefulness,* published in 1939, relied on a bibliography of 1434 references in several languages, only 11 of which were published before 1900.

Unfortunately, the study of the history of medical sciences does not attract many. In 1984, I spent a sabbatical in Alan Hobson’s laboratory at Harvard and spent many hours in the rare books section of its Countway Library, collecting pre-1900 literature on sleep and sleep disorders. On many occasions, I was the only visitor, thereby enjoying the full attention of the entire staff. They were happy to have a visitor. I found many gems among this vast collection of rare books and journals, which contributed to the materials I needed for several publications. I was delighted that some of my historic finds attracted wide attention. The two papers that described the early accounts of sleep apnea, published in the *Archives of Internal Medicine***[104]**, and in *Sleep Medicine Reviews***[105]**, were cited more than 100 times. When I submitted my first historical paper, “Nothing new under the moon,” to the *Archives*, I received a response from Dr, Alfred Soffer, the chief editor, within a few days. He wrote that he reviewed the paper himself and decided to accept it as is. It was the only time I have received such a response from an editor. The paper, “The origin of dream theories in the philosophy and physiology of the 18th and 19th centuries,” written together with Alan Hobson **[106]**, and the paper, “Rediscovering the importance of nasal breathing*”* [107], were each cited more than 50 times. Regretfully, my book *Brain, Structure and Function: Historical Concepts*, which has three editions, has been published only in Hebrew **[108]**.

## Open Questions

As is evident from my research history, my interests in sleep were not limited to only one subject. I was equally fascinated by dreaming, sleep apnea, and patterns of sleep propensity, it is logical to organize my career path into several distinct research areas. Occasionally, however, we conducted studies unrelated to these subjects, that we believed would be of special interest to the sleep community, particularly to young researchers looking for research ideas. I will mention only a few.

The first one is the fascinating case of Y.C., a young man referred to our clinics because of violent shouting during sleep **[109]**. Y.C. sustained a war-related head injury in the 1970s. He recovered, graduated from law school, and practiced law. Other than having been confined to a wheelchair and experiencing several other motor limitations, he led a normal life. The sleep laboratory findings were astonishing. In eight whole-night PSG recordings, Y.C. averaged only 2.25% of REM sleep. In three nights, there was no REM sleep whatsoever; on the other nights, there was a total of no more than 15 minutes of REM sleep, which appeared toward the end of the sleep period. Only on one night was there an episode of shouting occurring, as expected, in stage 4. At that time, two Canadian sleep researchers, Eliot Philipson and Harvey Moldovsky, were staying with us. I recall asking them to re-score the PSG records to make sure that I had not missed any REM period. They were astonished as well. The unexpectedly low amount of REM sleep prompted us to perform a brain CT scan, which had not been done before on this patient. The CT revealed a tiny metal splinter in the left pontine brain stem, at precisely the same location that Michel Jouvet had located the executive mechanism of REM sleep in cats **[110]**. This was the first documentation of a localized brain stem lesion associated with almost complete absence of REM sleep, in a person conducting a relatively normal life. This outstanding observation was recognized by the 1993 *Guinness Book of Records*.

Thirty-five years later, in collaboration with Yuval Nir’s group from TAU, we reevaluated Y.C. and confirmed the near-total absence of REM sleep. There were no signs of significant compensation throughout his adult life, along with normal cognitive status **[111]**. No doubt, the case of Y.C. challenges the prevailing views attributing an adaptive role for REM sleep in supporting cognition and memory. Disappointingly, it attracted only moderate interest from the sleep community (168 citations).

Another paper described a study related to my earlier studies on post-REM and non-REM awakening effects on perception. In 1982, we expanded this paradigm to test differences in cognitive dominance after awakenings from REM and non-REM sleep. We found that REM awakenings were associated with a right hemisphere superiority, and conversely, non-REM awakenings were associated with a shift toward left hemisphere superiority **[112, 113]**. These intriguing findings, combined with reports on a functional disconnection between the two brain hemispheres during REM sleep [114], may have important implications for the control of dreaming and dream formation. This area of research is awaiting an ambitious young researcher looking for an exciting challenge.

## Sharing Knowledge With Society

Universities are often described as intellectual ivory towers where scientists happily live in a world of ideas, apart from the realities of ordinary people’s lives. Although some scientists indeed reside in such ivory towers, I firmly believe that scientists should contribute to their institutions and to society and share their knowledge with the public. This has been my attitude even before I was elected President of the Technion. Thus, in addition to my career as a scientist in which I spent long days—and in my case, more so long nights—in the laboratory, I made efforts to contribute to my institution by taking on administrative and leadership positions. Over the years, I have had many such roles. I served as a chairman of a Technion research center (two terms); chairman of a department; was Dean of Medicine for 6 years; Vice President for Development for 7 years; and President of the Technion for 10 years. Until my appointment as President, I never slowed my research activity, in large part because I was lucky that Lena, my wife and research partner, ran the laboratory and brilliantly supervised the research. However, during my 10 years in the presidency, the responsibility of running the institution was overwhelming. I suppose I functioned well, given that I was the only President in the Technion’s 100-year history elected for three terms.

I am gratified that I made some contributions to the sleep research community. I was twice a member at large of the APSS executive committee (1983–1985; 1987–1990). I founded the Israeli Sleep Research Society and served as its chairman for 10 years. I served as a Vice President of the European Sleep Research Society (1988–1989) and, from 2008 until my appointment as the Technion President, I served as the Editor-in-Chief of the *Journal of Sleep Research* (JSR).

**Figure 2.**
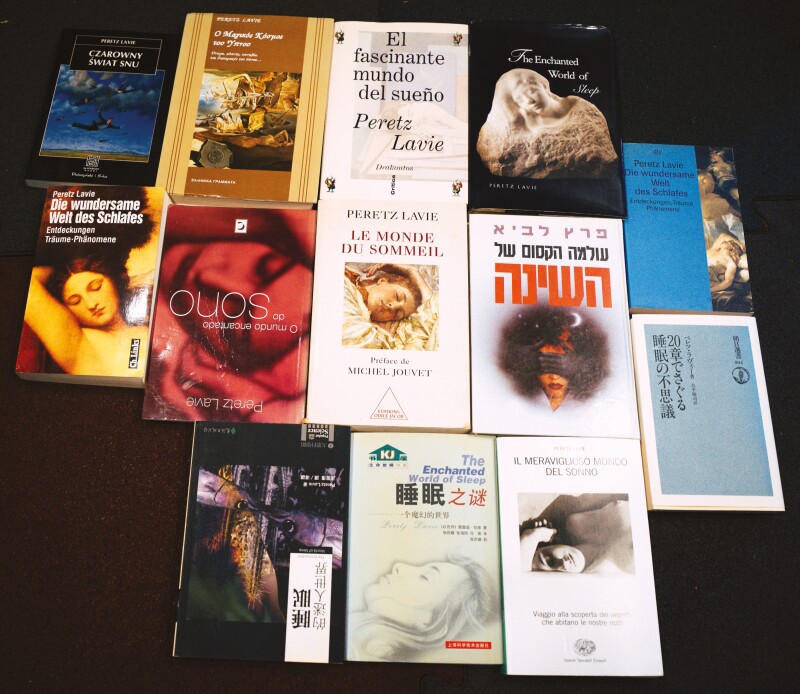
The Enchanted world of sleep, or how to say “good night” in fourteen languages (Credit: Peretz Lavie)

I also believe strongly that sharing knowledge with the public is an obligation of a university faculty member. To serve the public, we opened the Technion Sleep Clinics under the directorship of Dr. Ron Peled, who has directed this clinical service for more than 40 years. A record number of more than 150,000 patients have slept in our clinics; this may be a world record for a clinic! We were also instrumental in opening a similar service in Boston. In collaboration with Brigham and Women’s Hospital, we provided the model for opening its Sleep Health Center under the directorship of my friend and colleague David White. My first book, *The Enchanted World of Sleep***[101]**, was written for the public to spread knowledge about sleep. It did so in 14 languages! (Figure 2) *Sleep Disorders*, written with Giora Pillar and Atul Malhotra, was intended to educate physicians about sleep disorders **[115]**.

Some of our research results were conveyed to the public in the form of government policy decisions such as abolishing the “zero hours” in Israeli elementary schools (a class held before the formal opening of the school day), supporting public campaigns to prevent sleep-induced accidents of young drivers, and regulating the length and organization of night shifts in industry and hospitals. During the 1992 Gulf War in Israel, we convinced the radio authorities to open a “silent” radio channel. It was activated only in the case of a missile attack during the night and allowed people to sleep peacefully knowing that they would be awakened in case of an attack at night. Using actigraphy, we monitored sleep during wartime in that war, documenting the effects of night missile attacks on sleep in children **[116]** and adults **[117]** and reassuring the public about their sleep.

Being part of a technical university provided us with acquiring the knowhow to improve our sleep-monitoring devices and developing new ones. My technologically talented longtime assistant, Jacob Zomer, built new respiratory belts resistant to noise. Later, these belts were the first product of SLP, an Israeli company that produces a variety of sensors for the sleep community. We took part in introducing actigraphy to monitor sleep–wake cycles. The first algorithm to score infants’ sleep–wake cycles from actigraphy was developed by Avi Sadeh, as part of his PhD dissertation **[118, 119]**. Avi, one of my most brilliant students, became a world leader in pediatric sleep research. Avi died prematurely in 2016.

In 1997, a research project on peripheral blood flow during sleep led by my postdoc Dr. Bob Schnell **[120]** led to the development of the original peripheral arterial tonometry probe (the PAT). This was the basis for the start-up company Itamar Medical that produced and commercialized the now-popular Watch-PAT device **[121–123]**.

## Epilogue

In October 2019, I retired from the Technion’s presidency after serving for a decade, and Lena retired as well. We closed the Technion Sleep Research Laboratory, although our sleep clinic continues operating. Sleep research, particularly sleep medicine, has experienced exponential growth in the 50 years since I made my first sleep recording in TAU. The number of scientific papers on human sleep increased enormously. In 1968, not a single paper mentioned the term sleep apnea; 10 papers were published on Pickwickian syndrome, none of them in English. More than 3000 were published in 2022. Being part of this impressive growth is indeed a privilege, and having an H index of 103 and more than 35,000 citations (Google Scholar) suggests that I made a contribution to the field. It is gratifying that my contributions were recognized by the scientific community. In 2004, I was bestowed the University of Pisa Sleep Award, and in 2006 I was honored to receive the Prime Minister EMET prize, given for excellence in academic and professional achievements. In 2018 I was bestowed the UF Distinguished Alumnus Award, and in 2020 the Ministry of National Education of the French Republic awarded me the title “Commadeur dans l’Ordre des Palmes academiques” (Order of Academic Palms), which is a national order honoring academics for valuable service to universities, education and science, originally established in 1808 by Napoleon.

Another reason for looking back with a sense of accomplishment and satisfaction is the achievements of my students. They deserve recognition. Dr. Ron Peled was one of the first non-Americans to become a certified sleep physician and has been the head of the Technion Sleep Clinics since its beginning. He has seen more sleep patients than anybody in the world. Dr. Carlos Gordon (1981) was an Associate Professor of Neurology at TAU; Dr. Yaron Dagan (1990), an Associate Professor in the Department of Biology at Haifa University, and head of the Assuta Sleep Disorders Center; the late Avi Sadeh (1990), a Professor and Director of the Clinic for Children’s Sleep Disorders in the Department of Psychology at TAU, and Orna Tzishinsky (1993) and Iris Haimov (1995), both professors at the Max Stern Yezreel Valley College; Giora Pillar (1994), a Professor in the Technion’s Faculty of Medicine and Chair of Pediatrics at Carmel Hospital; Tamar Shochat (1997), a Professor and Chair of the Cheryl Spencer Department of Nursing at Haifa University; and Nir Peled (1998), Professor and Head of Shaarei Zedek Hospital Cancer Center in Jerusalem. Many of the physicians who spent research time in our sleep laboratory have become chairmen of clinical departments.

I am often asked how someone with so many responsibilities is able to be an active researcher. The answer is simple: It all depends on the people around you. I was blessed with an outstanding group of dedicated collaborators, assistants, and graduate students who were “infected” with the “sleep research virus.” They did most of the work. I could not have done it without them and without Lena, my partner and wife for 52 years.

I believe that the Talmudic expression, “I learned a lot from my teachers, and from my friends—more than my teachers, and from my students—more than anyone,” is an appropriate conclusion to my voyage in the enchanted world of sleep.
